# ReaDySpeech for people with dysarthria after stroke: protocol for a feasibility randomised controlled trial

**DOI:** 10.1186/s40814-017-0169-0

**Published:** 2017-07-20

**Authors:** Claire Mitchell, Audrey Bowen, Sarah Tyson, Paul Conroy

**Affiliations:** 1 Division of Neuroscience and Experimental Psychology, Faculty of Biology, Medicine and Health, University of Manchester MAHSC, Manchester, UK; 2 Division of Nursing, Midwifery & Social Work, University of Manchester, MAHSC, Manchester, UK; 3 Central Manchester University Hospitals NHS Foundation Trust, MAHSC, Manchester, UK

**Keywords:** Dysarthria, Stroke, Speech/language therapy (SLT), Feasibility, Randomised controlled trial

## Abstract

**Background:**

Dysarthria, a disordered speech production resulting from neuro-muscular impairment, is a common symptom after stroke. It causes significant problems for patients’ speech intelligibility, communication, psychological well-being, social engagement and stroke recovery. Rehabilitation for dysarthria is variable in quality, intensity and duration, which may be, in part, due to the lack of good quality evidence. An online therapy programme, ReaDySpeech, has the potential to improve quality, intensity and duration of speech rehabilitation and was considered in a proof-of-concept study to be acceptable to speech and language therapists and patients which warranted further evaluation. The present study aims to examine the feasibility of running a trial using the ReaDySpeech intervention.

**Methods/design:**

A feasibility, randomised controlled trial, will recruit a minimum of 36 people with post-stroke dysarthria who are more than 1 week post stroke. Participants will be externally randomised in a 2:1 ratio to receive either ReaDySpeech and usual care (24 participants) or usual care only (12 participants). This study is single blind with the researcher carrying out the baseline and outcome measures while blinded to treatment allocation. The primary objective is to assess the feasibility of conducting a larger Phase III trial. The specific objectives are to determine the following: recruitment rate and reasons for non-recruitment; loss of participants to follow-up; acceptability of randomisation; adherence to the intervention; delivery of ReaDySpeech and content; acceptability of outcome measures; success of blinding strategies; defining ‘usual’ care; and the implications of the intervention for the patient/family/carer.

**Discussion:**

This study will involve a regional, multi-centre, randomised controlled feasibility trial of a complex intervention in order to evaluate whether a Phase III randomised controlled trial is feasible.

**Trial registration:**

Current Controlled Trials, ISRCTN84996500

**Electronic supplementary material:**

The online version of this article (doi:10.1186/s40814-017-0169-0) contains supplementary material, which is available to authorized users.

## Background

Stroke is the second leading cause of death worldwide [1], and approximately 20–30% of stroke survivors [2] will experience dysarthria. Dysarthria following stroke has been found to have a negative impact on functional recovery, psychological well-being, social engagement and participation [3–5]. Dysarthric speech is less intelligible than that of healthy individuals due to poor control of oral articulator muscles, particularly the tongue and lips and poor respiratory control. Dysarthria affects people in many different ways depending on which muscle groups are impaired such as unclear articulation of words, nasal speech or a quiet voice with no expression for example. This variation in presentation also includes a wide severity range with some patients having no useful speech while at the milder end speech is generally intelligible but there may be lapses in speech accuracy or fatigue. The extent of disability also varies according to an individual’s communication demands, such as work and social situations, where mild dysarthria can be hugely disabling.

The evidence base regarding treatment for dysarthria after stroke is limited by a lack of adequately powered, well-controlled trials. A Cochrane review found no trials [6], and a more recent update found that while five trials could be included these were considered low to very low in quality [7]. These more recent randomised controlled trials are inconclusive about which intervention for dysarthria rehabilitation is most effective [8–12]. Thus, further high-quality trials are needed to benefit people with dysarthria given the potential for depression, social exclusion, and worse quality of life [4, 5, 13]. Researchers and therapists continue to evaluate and seek guidance about which intervention works best for dysarthria post stroke and what frequency and duration of intervention will give the best outcomes [13]. There is growing interest in the use of computer technology to help patients access therapy for dysarthria after stroke and, in doing so, enhance the individualisation and intensity of treatment delivery as well as ensuring choices are available so patient-centred care is accessible. Using technology to support dysarthria intervention could be cost-effective and enhance clinical and patient-reported outcomes from rehabilitation after stroke.

This paper summarises a protocol for our feasibility trial of the online programme ‘ReaDySpeech’ for people with dysarthria, accessed via any Wi-Fi enabled device. In terms of the ICF (International Classification of functioning, disability and health [14]), the ReaDySpeech programme addresses dysarthria impairment (improving speech musculature) as well as activity (compensatory strategies) levels. This programme was initially developed with exploratory interviews with speech and language therapists and patients [15], and then further enhanced by input from a research advisory group ‘Ever-ready’, made up of patients who have experienced speech problems after stroke. ReaDySpeech can be used in the acute and chronic stages of recovery and can be delivered alongside work to address participation. Proof-of-concept testing found ReaDySpeech to be acceptable to therapists and patients when used in a clinical context [15]. This protocol is the next step in evaluating ReaDySpeech. Here we describe a Phase II feasibility trial which will enable us to determine the feasibility of carrying out this research on a larger scale [16, 17]. This protocol has followed the CONSORT (Consolidated Standards of Reporting Trials) guidelines [18] and the SPIRIT (Standard Protocol Items: Recommendations for Interventional Trials) statement [19] as well as the TIDieR (Template for Intervention Description and Replication) checklist and guide [20].

### Aim

The primary objective is to assess the feasibility of conducting a Phase III randomised controlled trial comparing ReaDySpeech with usual care versus usual care only.

Study objectives: This study aims to determine the following:

1. Number of participants eligible for the study, recruitment rates and reasons for declining, retention rates and reasons for loss of patients for future trial sample size
2. Delivery of ReaDySpeech and content selection
3. Adherence to the technology in the intervention arm
4. Content of ‘usual’ care: activities, intensity, duration
5. Clinical utility and acceptability of outcome measures to patients and effectiveness of blinding
6. Impact of intervention on patient, family/carer/partner

## Methods/design

### Study design

This is a feasibility, single-blind, individually randomised controlled trial of ReaDySpeech with usual care versus usual care.

### Setting

This is a multi-centre study recruiting from four NHS sites in North West England over 14 months. Recruitment and treatment will take place in hospital and community locations including patients’ homes.

### Population

The study population includes adults (aged ≥18 years), with dysarthria as a result of stroke.

### Entry criteria for participation in the trial

Inclusion criteria are as follows:

1. Diagnosis of dysarthria caused by stroke
2. More than 1 week post stroke, no upper time limit
3. Medically stable, as judged by the clinical team
4. Considered, by their speech and language therapist, to be likely to benefit from speech rehabilitation
5. Sufficient English to participate in therapy without a translator

Exclusion criteria are as follows:

1. Co-existing progressive neurological conditions
2. Co-existing communication, cognitive, hearing or visual problems significant enough to prevent use ReaDySpeech

### Identification and recruitment of trial participants

Potential participants will be identified by NHS speech and language therapists in the participating stroke services from their patient caseloads including hospital and community settings. The ReaDySpeech study screening log will be completed by the speech and language therapists to record the number of dysarthric stroke patients who are not eligible for the study and the reasons why, and the number of those who are eligible for the study but decline participation including their reasons if provided. The speech and language therapists will identify patients meeting the inclusion criteria, give out the patient information sheet to those willing to find out more, and inform the researcher (with the potential participant’s agreement). The researcher will contact the patient once they have had 24 h to read the study information and answer any questions. If the participant meets the inclusion criteria and is willing to participate, the researcher will obtain signed, fully informed consent in line with Research Ethics Committee guidance and Good Clinical Practice Standards. Baseline assessments will be carried out at this point prior to randomisation and intervention will start immediately after allocation.

### Randomisation

Participants will be randomised using a 2:1 allocation ratio to ReaDySpeech and usual care (intervention arm) or usual care only (control arm) respectively. The primary researcher will enter minimal anonymised patient details onto the external web-based randomisation programme held by an independent clinical trials unit to ensure allocation concealment. The programme will generate an un-blinded email to the treating therapist, who will inform the patient of their treatment allocation when intervention starts. Minimisation will be stratified by the four sites and by acute stroke (≤12 weeks post stroke) or chronic stroke (≥12 weeks post stroke).

### Blinding

Given that rehabilitation involves active participation, it is not possible for the participants and treating therapist to remain blinded to the treatment allocation. The primary researcher will be blinded to the intervention allocation and ask the speech and language therapists delivering the intervention and the patients or family to not to reveal the treatment allocation. They will be reminded of this at the start of every interaction. The primary researcher will remain blind until after the outcome measures have been carried out at 8/10 weeks post randomisation. At that point, the researcher will record which group they thought the patient was in to look at the effectiveness of the blinding process as well as documenting any reasons where blinding was affected.

### Sample size justification

No formal sample size calculation was carried out. Thus, the sample size for this feasibility trial was governed pragmatically by the resources available. Based on our experience of undertaking communication-related trials, we decided to recruit for 14 months, from four NHS sites in the North West of England, estimating this would provide around 36 participants. This will give us an indication of the expected variability of service delivery, resource availability and produce more generalisable recruitment and retention rate. Recruitment will be reviewed 4 months into the study to whether the recruitment strategy needs to change. The 2:1 allocation provides a larger group with whom to explore intervention delivery and fidelity. It means the smaller group would have a minimum sample of 12 which is considered acceptable for a feasibility study [21].

### Description of the intervention

#### ReaDySpeech

ReaDySpeech is an online programme to deliver dysarthria therapy at impairment and activity levels of functioning for people following stroke. The intervention is described in detail in Additional file 1 following the TIDieR checklist [20]. ReaDySpeech will record what exercises were prescribed by the therapist and which were completed by the patient.

#### Usual speech and language therapy care

Usual speech and language therapy will be accessed by those randomised to the control group as well as the ReaDySpeech arm. This would be expected to follow existing best practice guidelines which address impairment, activity and participation levels of functioning [22] and is described in detail in Additional file 1 following the TIDieR checklist [20]. The frequency, duration and content of the sessions will be extracted retrospectively from the clinical speech and language therapy notes by the primary researcher in partnership with the therapist.

#### Assessment of objectives

Feasibility will be determined by the recruitment and retention rates found at the four sites over the 14-month trial. This will enable us to look at the sample size needed and the number of sites required to recruit to a Phase III trial from a formal power calculation. Data on reasons for exclusion and eligible participants declining involvement in the study will also help to assess whether this study is feasible for a larger population and whether recruitment should be amended in any way.

Fidelity to look at how ReaDySpeech was delivered will cover delivery, access and support. We will record who delivered ReaDySpeech, whether this was independent use, therapist, assistant or family led; whether computers were loaned to participants and whether participants were supported and trained to use the programme. Adherence data will also be examined to assess participants’ use of ReaDySpeech. The programme software will record exercises selected by the therapist and which of these exercises are recorded online as having been completed by the participant. The usual care provision adherence data will be taken from the clinical case notes and will allow us to describe current provision of ‘usual’ speech and language therapy in the four participating sites with a potential to formulate what this could look like in a future trial.

Following completion of the outcome measures, patients will be asked structured and open questions by the primary researcher in a face to face interview, and answers will be written down. Interviews in our previous acceptability work found responses were brief and written documentation sufficient. The questions will explore four key areas: (i) what the participants thought about the research study, whether they understood the study and what was going to happen, their views on randomisation, whether the time taken for the outcome measures was acceptable and whether they felt the outcome measures reflected their views of their speech; (ii) what was delivered in ‘usual’ care and/or ReaDySpeech from their perspective including who delivered it and when; (iii) the impact of usual care or ReaDySpeech on themselves, family, partner, carer; (iv) any other comments on the study and/or the interventions for the research team to consider.

This will enable the research team to consider the implications of participating in this research and participant’s views of the study itself as well as the acceptability of the outcome measures.

##### Baseline measures

Demographic data (age and gender), stroke information (time since stroke, type of stroke–haemorrhagic or infarction and stroke classification), levels of pre-morbid and current functioning (modified Rankin Scale [23]) and current activities of daily living (Barthel Index, 10-item scale with 5-point increments, based on Mahoney and Barthel’s tool [24–26]) and the co-existence of other language impairments such as aphasia (severity, how it was diagnosed) will be extracted from notes and documented prior to randomisation. Measures completed at baseline, prior to randomisation, will be therapist reported speech at activity level (Dysarthria Therapy Outcome Measure, TOMS Activity score [27]); patient reported communication at activity and participation level (Communication after Stroke Scale, COAST [28]); patient-reported communication at activity and participation level (Dysarthria Impact Profile, DIP [29]); therapist reported speech at impairment level (Frenchay Dysarthria Assessment 2nd edition, FDA II [30]); and a patient-reported health outcome measure (EQ-5D-5L [31]) that may provide useful feasibility data for a future cost-effectiveness study.

##### Outcome measures

The measures carried out at baseline will also be carried out at the end of intervention (8/10 weeks post randomisation) by the primary researcher either in hospital or the participants home (if they have been discharged). Change from baseline scores will be examined to determine the sensitivity of the measures.

#### Assessment of blinding

To assess the effectiveness of the blinding, the primary researcher will guess group allocation for each participant once the outcome measures have been completed. They will then check this guessed allocation against the randomised allocation record to examine effectiveness of blinding when the database is locked and the code un-blinded.

#### Data management

Personal data, case report forms and participant questionnaires will be treated as confidential documents and held securely in accordance with the NHS research ethics committee regulations as outlined in the ethics approval process. Each consenting participant will have a unique identifier that will be used for randomisation and identification. The externally held randomisation programme at the clinical trials unit will have no identifying patient information and all data entered on the programme will be stored securely in accordance with the standard operating procedures at the clinical trials unit. There will be no dates of birth or NHS numbers recorded during this study, only age. Time since stroke will be recorded, not the date of the stroke. A data monitoring committee was not deemed necessary for this small feasibility trial given the intervention (online speech rehabilitation) is so low risk and not expected to lead to serious related adverse events.

#### Data analysis

Analysis for this single blind, multi-centre, feasibility randomised controlled trial will be descriptive as the study is not designed to look at the effect of the intervention and would not have sufficient statistical power.

**Fig. 1:**
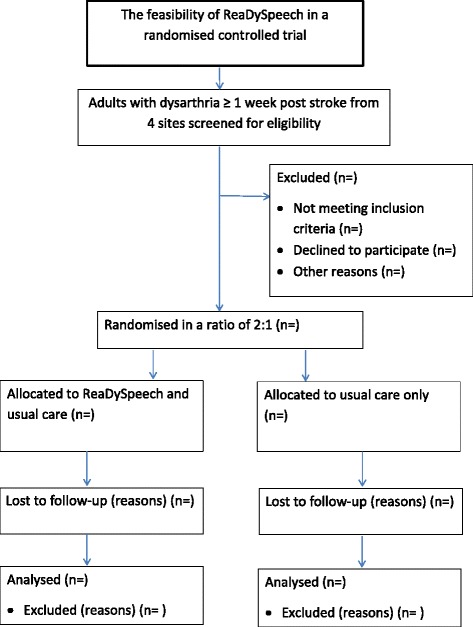
ReaDySpeech participant flowchart through trial

Descriptive summary statistics will consider the numbers of patients who were eligible, recruitment to the study and attrition rates according to site and intervention arm. A CONSORT (Consolidated Standards of Reporting Trials) and SPIRIT (Standard Protocol Items: Recommendations for Interventional Trials) flow chart will present the overall recruitment to the study [19] (Fig. 1).

Demographic data will be examined to identify if there are any patterns of recruitment related to age, gender, stroke type, severity of disability or health, whether the randomised groups are balanced at baseline and whether this recruited sample is reflective of the stroke population. We will consider change between baseline and follow-up measures to consider possible effect sizes of the potential primary outcome measure for the future trial. The recruitment and retention rate data will support the sample size calculation for a future larger trial and the likely number of sites needed to achieve this sample. No analysis of outcome measures will be undertaken until all follow-up assessments have been completed. Both the ReaDySpeech and usual care therapy provided will be described including fidelity and adherence data and interview data [20] (Additional file 1).

#### Safety monitoring and adverse events

A risk assessment using the technology as part of this trial indicates that the risk of harm is low. The proof of concept work also showed it was safe [15]. Any previously un-identified risks of the experimental intervention will be documented and reviewed with the study sponsor. The study can be audited at any point by the funders, sponsor or REC, and documentation for this purpose will be maintained in a study master file.

## Discussion

This study will provide evidence for the feasibility of a randomised controlled trial into the effectiveness of ReaDySpeech for people with dysarthria after stroke. The qualitative and quantitative data produced will inform the decision about the potential for a subsequent trial. The paucity of existing randomised controlled trials of interventions for dysarthria after stroke means that the findings will also be of interest to other researchers working in this area or wanting to examine recruitment for other technology studies in similar populations. The likely variations in usual care will be an additional finding from this research that will be of interest to both researchers and clinicians.

This feasibility trial’s findings will be presented at national and international stroke and rehabilitation conferences and submitted for publication in peer-reviewed journals. They will also be disseminated to stroke survivors and study participants in a user-friendly format which will be produced in partnership with our research advisory group ‘Ever-ready’. Finally, the results will also be disseminated through social media.

### Trial status

Participant recruitment started in September 2015 and is due to finish recruiting by the end of October 2016. The trial is registered with ISRCTN84996500.

## Additional file

Additional file 1: Description of ReaDySpeech and usual care (TIDieR). (DOCX 18 kb)

## Abbreviations

CTU: Clinical trials unit
Dysarthria TOMs: Therapy outcome measure specific to dysarthria
ISRCTN: Numerical identification of randomised controlled trials
NHS: UK National Health Service
RCT: Randomised controlled trial
ReaDySpeech: Online programme for the rehabilitation of dysarthric speech
SLT: Speech and language therapy or therapists
