# Supplementary material for: ReaDySpeech for people with dysarthria after stroke: protocol for a feasibility randomised controlled trial
Source: Pilot Feasibility Stud. 2017 Jul 20;4:25. doi: 10.1186/s40814-017-0169-0 (PMC5520339; doi:10.1186/s40814-017-0169-0)
Supplement: Additional file 1: — Description of ReaDySpeech and usual care (TIDieR). (DOCX 18 kb) [file 40814_2017_169_MOESM1_ESM.docx]

**Template for Intervention Description and Replication for the ReaDySpeech intervention**

- ReaDySpeech is an online programme which delivers exercises and strategies to improve intelligibility for people with dysarthria after stroke.
- This intervention is based on existing best practice guidelines which address impairment and activity levels of functioning.
- The exercises are selected by a speech and language therapist (slt) for each individual patient to be accessed via the ReaDySpeech online programme <https://amie-test.herokuapp.com/>. This can then be accessed with a username and password.
- The slt will select the intervention according to clinical need when considering the duration, intensity or dose of intervention as agreed with the patient.
- The programme will be adapted to the patients progress.

The activities used in the intervention include;

- - Practising articulation of
    - specific sounds in isolation
    - words of increasing syllable length
    - words with specific sounds in word initial position
    - words with specific sounds in word final position
    - complex clusters in word final position
    - short phrases and questions to repeat or answer
  - Breathing exercises
    - maintaining breath support
    - controlled breathing
    - breathing and speaking
  - Practising rate of speech and intelligibility strategies using an alphabet chart or a pacing board
  - Volume work
    - word level
    - speaking in short sentences
  - Facial expression
  - Intonation exercises
    - sentence level
  - Oro-motor exercises to improve range of movement, strength and speed of movement.
- All the activities appear on screen in written format and show progress, the oro-motor exercises include video clips and verbal instructions for each one.
- The intervention is used by the individual independently or with support from family, therapy assistant or speech and language therapist. There is no required level of training to support this intervention.
- The intervention is provided via the internet on any Wi-Fi enable device.
- The intervention could be accessed in any setting that has Wi-Fi access, for this study that could be in the acute or rehabilitation in-patient setting or the community in a patients home, residential or nursing home setting. If Wi-Fi access is not available this could be provided via a Wi-Fi enabled device using a paid for sim card.

**Template for Intervention Description and Replication for ‘USUAL CARE’**

- Usual speech and language therapy for dysarthria is based on Royal College of Speech and Language Therapy Clinical Guidelines [1] to deliver intervention at impairment, activity and participation levels of functioning. The guidelines are based on the best available evidence from research trials, case studies and expert opinion.
- Usual care is delivered by face to face sessions with a speech and language therapist or therapy assistant. Paper based materials describing exercises and including words/sentences to practice are used during the sessions and left with the patient for independent practice.
- Impairment level therapy focuses on exercises for function, strength, speed and precision of impaired musculature. This can also include: breathing exercises, work to improve resonance, phonation, articulation and prosody. Activity level therapy addresses compensatory approaches to speech such as reducing rate of speech, enhanced articulation, as well as environmental modifications and augmentative approaches such as an alphabet chart or text-to-speech aids. Participation level approaches involve education, psychological support, working with conversation partners and other person-centred approaches to support individuals in their work and life following stroke.
- Usual care is delivered by speech and language therapists, assistants or with family support according to the speech and language department’s usual model of service delivery and responsive to patient need.
- Usual care is delivered in acute and rehabilitation in-patient settings and in any community setting.
- No specifications were given to therapists delivering usual care regarding the duration, intensity or dose of intervention other than to use it according to clinical need as agreed between therapist and patient. Usual care is expected to be responsive to patient need and variable between patients and departments according to service delivery.

1. Taylor-Goh S: *Royal college of speech & language therapists clinical guidelines.* Speechmark; 2005.
